# Head model dataset for mixed reality navigation in neurosurgical interventions for intracranial lesions

**DOI:** 10.1038/s41597-024-03385-y

**Published:** 2024-05-25

**Authors:** Ziyu Qi, Haitao Jin, Xinghua Xu, Qun Wang, Zhichao Gan, Ruochu Xiong, Shiyu Zhang, Minghang Liu, Jingyue Wang, Xinyu Ding, Xiaolei Chen, Jiashu Zhang, Christopher Nimsky, Miriam H. A. Bopp

**Affiliations:** 1 Department of Neurosurgery, University of Marburg, Baldingerstrasse, 35043 Marburg, Germany; 2 Department of Neurosurgery, First Medical Center of Chinese PLA General Hospital, 100853 Beijing, China; 3 Medical School of Chinese PLA General Hospital, 100853 Beijing, China; 4 NCO School, Army Medical University, 050081 Shijiazhuang, China; 5 Department of Neurosurgery, Division of Medicine, Graduate School of Medical Sciences, Kanazawa University, Takara-machi 13-1, 920-8641 Kanazawa, Ishikawa Japan; 6 Center for Mind, Brain and Behavior (CMBB), 35043 Marburg, Germany

**Keywords:** Translational research, CNS cancer, Brain imaging, Magnetic resonance imaging, Neurosurgery

## Abstract

Mixed reality navigation (MRN) technology is emerging as an increasingly significant and interesting topic in neurosurgery. MRN enables neurosurgeons to “see through” the head with an interactive, hybrid visualization environment that merges virtual- and physical-world elements. Offering immersive, intuitive, and reliable guidance for preoperative and intraoperative intervention of intracranial lesions, MRN showcases its potential as an economically efficient and user-friendly alternative to standard neuronavigation systems. However, the clinical research and development of MRN systems present challenges: recruiting a sufficient number of patients within a limited timeframe is difficult, and acquiring low-cost, commercially available, medically significant head phantoms is equally challenging. To accelerate the development of novel MRN systems and surmount these obstacles, the study presents a dataset designed for MRN system development and testing in neurosurgery. It includes CT and MRI data from 19 patients with intracranial lesions and derived 3D models of anatomical structures and validation references. The models are available in Wavefront object (OBJ) and Stereolithography (STL) formats, supporting the creation and assessment of neurosurgical MRN applications.

## Subject terms

Mixed reality, Navigation, Neurosurgery, Intracranial lesion, Computed tomography, Magnetic resonance imaging

## Background & Summary

Intracranial lesions, pathological alternations within various brain regions, can exert pressure on critical neural structures^1^, potentially leading to neurological deficits or life-threatening conditions^2–4^. Thus, timely diagnostics and neurosurgical intervention is essential to preserve neurological functions, improve quality of life, and avert risks^2,5,6^.

Substantial advancements have been made in the methods by which neurosurgeons approach and treat intracranial lesions over the years. For instance, commercial neuronavigation systems have precisely transformed neurosurgical interventions by tracking the patient’s body and surgical instruments^7–9^. While these systems significantly enhance surgical precision, traditional techniques, such as pointer-based navigation, present ergonomic challenges^10–14^. Neurosurgeons frequently find themselves switching instruments, leading to disruptions, and they must toggle their focus between surgical site, navigation tools and monitors. This continuous shifting of attention and the mental effort required to integrate images into the surgical context can significantly increase cognitive load and mental strain, potentially affecting performance and learning in both surgical and educational settings^10–14^.

Augmented reality (AR) and, thereof, microscope-based navigation has emerged as a significant breakthrough. It employs the microscope’s optical focus as a virtual guide, superimposing digitally outlined structures directly onto the surgical field^8,9,15,16^. This reduces the need to shift attention and has proven clinically beneficial, enhancing comfort and understanding of anatomical structures. However, standard combined navigation and AR systems are expensive and require extensive procedural setup, prompting interest in more accessible alternatives. Among these, head-mounted device (HMD)-based AR, especially using optical see-through variants, stands out for its immersive and cost-effective nature^11–13,17,18^.

Shifting the focus to another technological advancement, Mixed Reality (MR), blending the physical and virtual worlds, offers an interactive environment distinct from AR^10,12–14,18–20^. MR technology digitizes real-world data, allowing more than overlaying virtual elements. Introduced to the market by Microsoft’s HoloLens, its advanced localization capabilities permit stable integration of three-dimensional (3D) elements into reality. The growth in mixed reality navigation (MRN) research highlights its potential as a cost-effective and user-friendly alternative appraoch to traditional neuronavigation systems^10–12,14,18,21–26^.

Essential to MRN’s functionality is the precise alignment of preoperative imaging data with the patient’s physical anatomy. This is achieved through various registration techniques, starting with procedures similar to those from conventional navigation systems, such as landmark-based^14,18,27–29^ and surface-based approaches^30,31^, and extending to manual alignment^12,13,20,32–34^ and registration based on a laser crosshair simulator (LCS)^21,35^. A straightforward, reliable, and minimally user-dependent registration method can boost the neurosurgeon’s confidence in using MRN^21,35^. On the other hand, MRN systems combining accurate anatomical and multimodal imaging data, such as blood flow information and white matter tracts, offer a holistic visualization, minimizing the risk of surgical complications and neurological impairment^18,36^. In summary, the virtual-physical alignment and the integration of diverse imaging modalities stand out as active fields in MRN research.

While testing MRN systems in clinical settings can directly validate their potential benefits for neurosurgical interventions, numerous challenges exist. Recruiting a sufficient number of patients to verify the clinical feasibility of a new technology often takes a long time^37^. Obtaining comprehensive data and informed consent from these patients within constrained timeframes poses additional challenges^37^. Furthermore, securing ethical approval for non-commercial medical device trials adds complexity and delays MRN development due to the rigorous documentation needed for safety and efficacy validation. Some researchers turn to commercially available patient head or skull phantoms, but these are costly. Everyday plastic phantoms serve as a cheaper alternative, but their medical relevance is limited^37^.

In this way, the contribution of this study to dataset construction is twofold. Firstly, a novel dataset tailored for MRN system development and testing in the neurosurgical domain is introduced. This dataset includes computed tomography (CT) or multimodal magnetic resonance imaging (MRI) data from 19 intracranial lesion patients. These data generated and optimized Wavefront Object (OBJ) files of anatomical structure holograms and Stereolithography (STL) files of the patients’ heads for cost-effective 3D printing. These models are invaluable for testing MRN registration algorithms and refining system functionalities before clinical testing. Secondly, a technical validation, ensuring the dataset’s validity and reliability is generated. This rigorous validation ensures that researchers can easily replicate and apply the findings to optimize their MRN systems, emphasizing the study’s significance and potential impact on the neurosurgical community.

## Methods

**Fig. 1:**
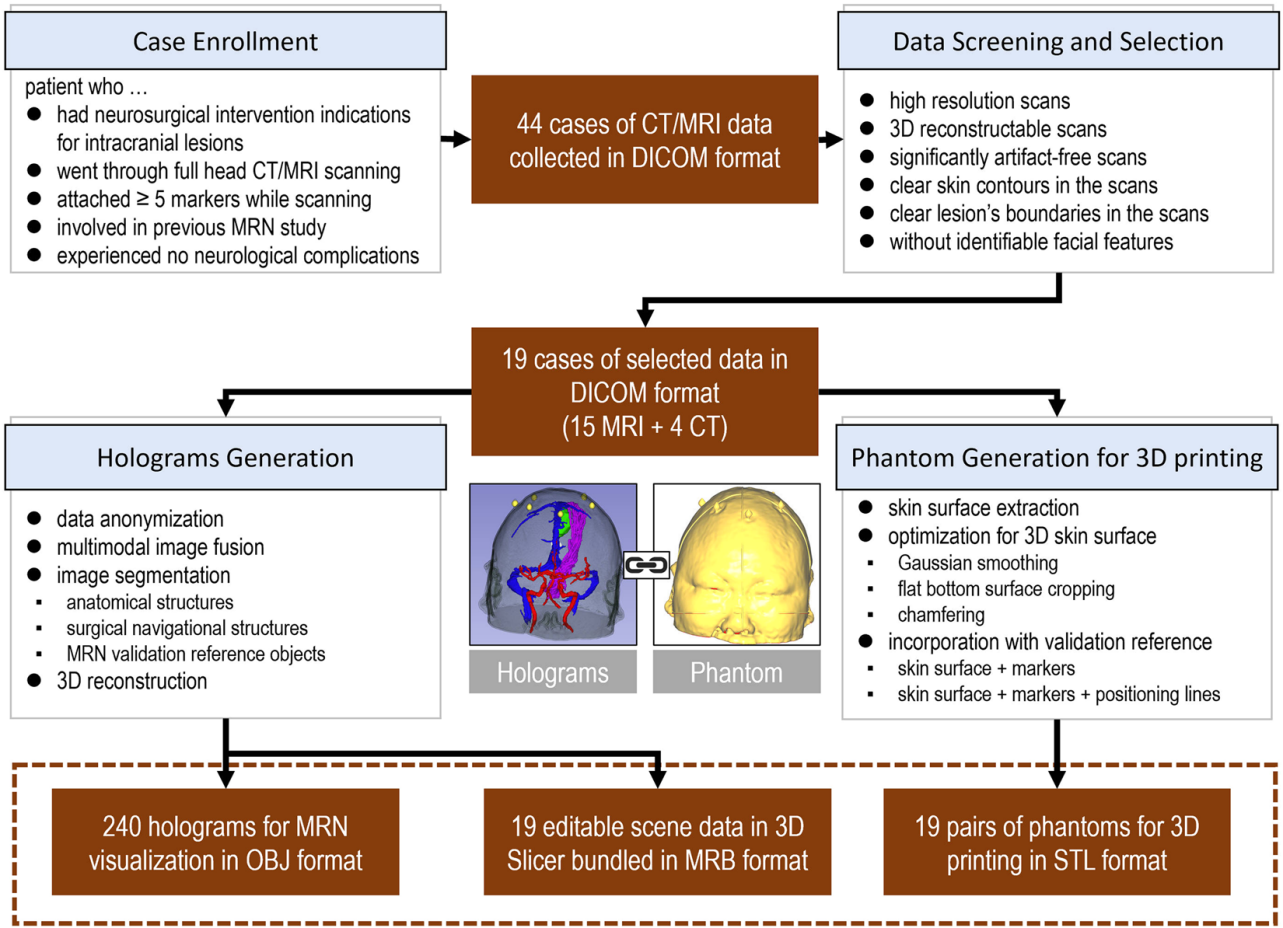
Practical workflow to produce the data in this study. Based on the enrollment criteria, CT/MRI data from 44 cases were collected in DICOM format. Undergoing a structured screening, data from 19 cases were chosen for further processing. On the one hand, the imaging data were reformatted, anonymized, segmented, and 3D reconstructed to generate holograms for visualization using MRN. On the other hand, the skin surface on each patient’s head was extracted and reconstructed from the data, then optimized for low-cost 3D printing and incorporated with validation reference objects. 3D = three-dimensional; CT = computed tomography; MRI = magnetic resonance imaging; MRB = medical reality bundle; MRN = mixed reality navigation; OBJ = object; STL = stereolithography.

This section outlines the construction process of the dataset, beginning with case enrollment and data selection (see Fig. 1). It proceeds through a sequence of image processing steps, including anonymization, de-identifiability, image fusion, segmentation, 3D reconstruction, and optimization, to generate 3D models that support holographic visualization and 3D printing tailored for testing MRN systems.

### Subject cohort

The study collected preoperative cranial MRI and CT data from 44 consecutive patients diagnosed with intracranial lesions, including neurological neoplasms and hypertensive cerebral hemorrhages, gathered over four years (2018–2021) in two facilities: the First Medical Center of Beijing and the Hainan Hospital in Sanya, both affiliated with the Chinese PLA General Hospital. With the approval of the Institutional Review Board (IRB) of the Chinese PLA General Hospital (Approval number: S2023–142–01), informed consent for using and publishing their potentially identifiable imaging data for research was obtained from each patient or their legal relatives, ensuring that data with uniquely identifiable characteristics were excluded for adequate de-identification to prevent privacy breaches, in accordance with ethical guidelines.

In all cases, more than five adhesive skin markers were attached to the scalp before imaging to establish known landmarks in the physical world within the images. As previously published by the study group^14,18,21,35^, these markers served as reference points for patient registration and comparing the MRN system with standard navigation systems. The surgeries, conducted under the guidance of standard navigation systems without significant complications, not only adhered to clinical routine standards requiring high-quality preoperative imaging to avoid complications but also served to validate the preoperative imaging and marker configuration. This dual role laid a solid foundation for evaluating the MRN system, and highlighted the data’s relevance and accuracy for assessing this innovative system, despite the surgeries not directly utilizing MRN system.

### Image acquisition

MRI data were acquired using a 1.5 T MRI scanner (Espree, Siemens, Erlangen, Germany), while CT data were collected with a 128 multislice CT scanner (SOMATOM, Siemens, Forchheim, Germany). The MRI scanning parameters were: T1-weighted imaging (T1WI) and T1-weighted contrast-enhanced (T1-CE) imaging using a magnetization prepared rapid acquisition gradient echo sequence (MPRAGE) with the administration of gadolinium (repetition time (TR) 1650 msec, echo time (TE) 3.02 msec, matrix size 192 × 256, field of view (FoV) 187.5 × 250 mm^2^, 176 slices, slice thickness 1.00 mm), T2-weighted sequence (T2WI, TR 5500 msec, TE 93 msec, matrix size 240 × 320, FoV 172.5 × 230 mm, 30 slices, slice thickness 3.90 mm), Diffusion tensor imaging (DTI) data using a single shot spin echo diffusion-weighted echo planar imaging (EPI) sequence (TR 9200 msec, TE 86 ms, matrix size 128 × 128, FoV 250 × 250 mm^2^, 40 slices, slice thickness 3.51 mm, no intersection gap, 20 diffusion-encoding gradient directions, high b-value 1000 s/mm^2^). The CT scanning parameters were: tube voltage 120 kVp, current 50 mA, window width 120, window level 40, matrix size 512 × 512, FoV 251 × 251 mm^2^, and slice thickness 0.625 mm resulting in a voxel size of 0.500 × 0.500 × 0.625 mm^3^.

### Data selection

To maintain the dataset’s integrity and homogeneity, the inclusion criteria for imaging data were stringent, necessitating high-quality, high-resolution imaging with visible intracranial lesion boundaries in at least one image sequence. Imaging data exhibiting significant artifacts or spatial distortion was excluded. Importantly, images lacking complete cranial or skin contours were also discarded, as they were unsuitable for generating comprehensive life-sized head phantoms for 3D printing. Additionally, given the critical role of patient facial features in the registration process for both standard navigation and MRN systems, no algorithms that could potentially modify the original imaging facial features were used. To protect patient privacy, de-identification procedures were applied at the case enrollment stage, involving a thorough examination of patient images to eliminate cases with identifiable facial anomalies or scars. Visual inspections by three independent neurosurgeons (Z.Q., X.C., and J.Z.) confirmed that all selected cases were non-identifiable by facial characteristics. Ultimately, data from 19 patients were chosen based on these criteria, with the remainder excluded. Among the selected patients (female / male: 7 / 12, mean age: 54.4 ± 18.5 years), 15 were subjected to MRI, and four to CT scans. The demographic information can be found in Table 1.

**Table 1:** Demographic metadata for cases included in the dataset.

No.	Sex	Age [years]	Histopathological diagnosis	Lesion volume [cm^3^]	Lesion depth [cm]
01	Male	49	Left temporal metastasis	5.2	2.8
02	Male	58	Right temporal diffused astrocytoma	8.4	8.4
03	Male	8	Left frontal cavernous malformation	13.8	4.1
04	Male	62	Left cerebellar meningioma	26.4	3.8
05	Male	41	Left occipital diffuse large B-cell lymphoma	16.5	4.0
06	Female	27	Left frontal meningioma	106.0	3.1
07	Male	51	Right occipital metastasis	48.9	4.1
08	Female	66	Bilateral occipital metastasis	Left: 3.2 Right: 10.7	Left: 2.9 Right:2.7
09	Female	37	Fourth ventricular aneurysmal change	1.4	5.7
10	Female	73	Right parietal metastasis	2.5	3.0
11	Female	54	Left parietal meningioma	10.1	3.9
12	Male	79	Left occipital metastasis	20.7	2.0
13	Female	41	Left parietal high-grade glioma	3.3	2.1
14	Male	74	Right frontal and occipital metastasis	Frontal: 7.5 Occipital: 1.1	Frontal: 2.2 Occipital: 2.7
15	Female	58	Left occipital metastasis	42.0	3.6
16	Male	84	Right frontal hemorrhage	73.5	4.0
17	Male	63	Right basal ganglia hemorrhage	33.9	4.0
18	Male	57	Right basal ganglia hemorrhage	34.0	5.3
19	Male	51	Left basal ganglia hemorrhage	30.0	5.3

### Data anonymization

Data preprocessing was performed using the freely available open-source software platform, 3D Slicer (Version 5.1.0, https://www.slicer.org/)^38^. Upon importing the data of the selected patients, the imaging sequences were initially converted from the Digital Imaging and Communications in Medicine (DICOM) file format to the Nearly Raw Raster Data (NRRD) file format, which was fully anonymized and stripped of the patient’s metadata. The transition to the NRRD file format ensured complete anonymization and enhanced data handling. Additionally, NRRD could maintain the integrity of the original imaging data without compression or damage, allowing for reconversion back to a metadata-free DICOM format when necessary, ensuring broad compatibility and adherence to privacy protection standards.

### Image fusion

**Fig. 2:**
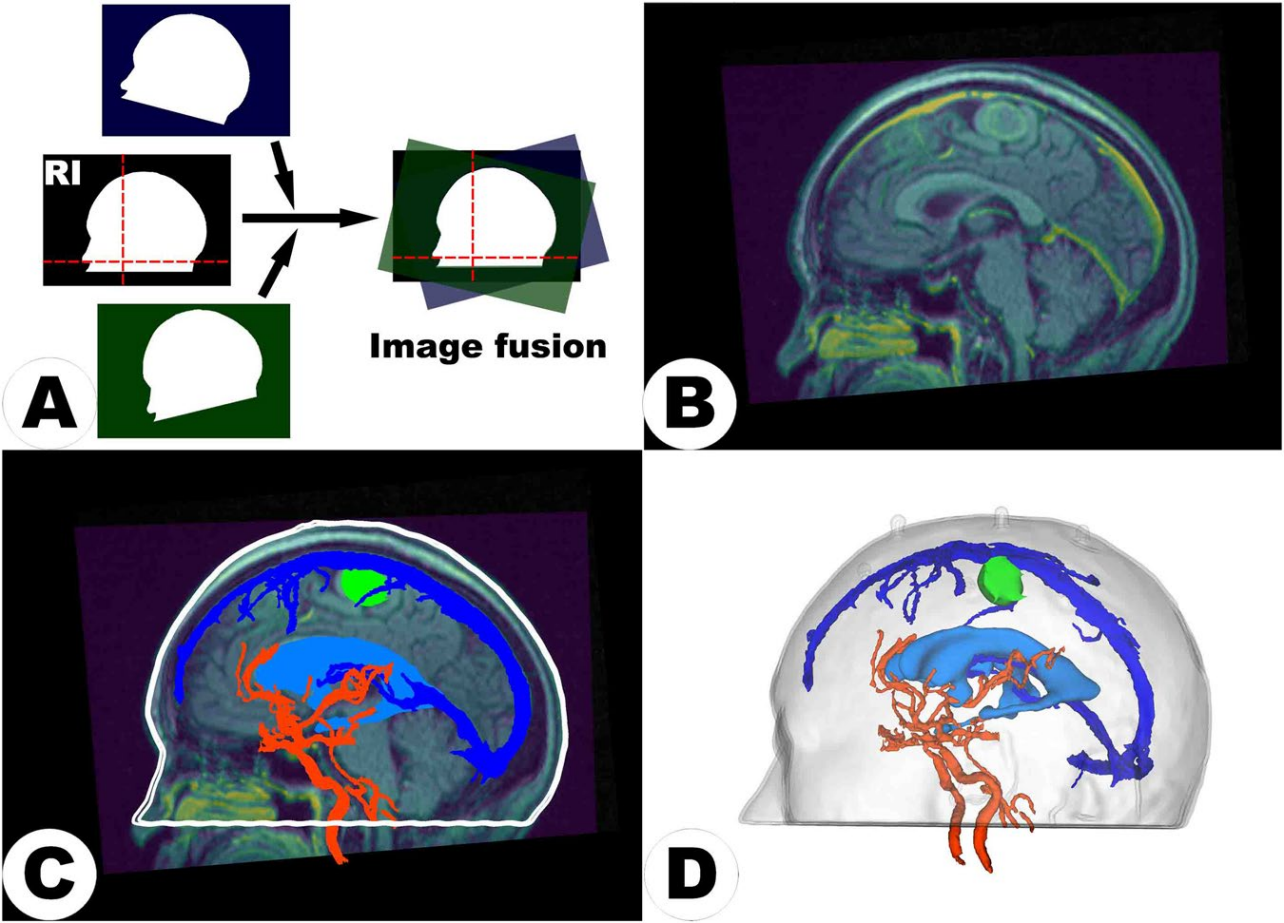
An illustration of the process of generating holograms. Subfigure (**A**) demonstrates the co-registration of a patient’s multimodal sequences into a unified coordinate system (indicated by the red dashed crosshairs), which is defined by the highest resolution reference image (RI). Following image fusion (**B**), synchronous observation is permitted, and segmentation is performed within the unified coordinate system (**C**). Subsequently, clusters of segmented voxels are transformed into a 3D surface model, i.e., holograms, which can be observed from any angle, not limited to the given imaging planes (**D**).

Neuroimaging data, if encompassing multimodal sequences acquired at various times, modalities, or scanners, required co-registration to amalgamate comprehensive information based on the multiple image input, thus aiding in precise surgical planning and functional preservation. This essential processing step aligned images from diverse modalities, such as T1-CE, T2WI and DTI, or images of the same modality obtained at different intervals. If imaging comprised only a single modality, such as cases of cerebral hemorrhage undergoing baseline CT scans alone, co-registration was not involved. The highest-resolution scan was used as the reference image (RI) to ensure accurate alignment (see Fig. 2A). It not only allowed the fusion of images for simultaneous observation and analysis (see Fig. 2B) but also harmonized their coordinate systems (i.e., aligning origins, orientations, and scales) to make various image-defined content such as segmentation, models, and trajectories visible, interactive, and modifiable across different images, ensuring a unified and precise integration of all data within a consistent coordinate system (see Fig. 2C). Each case’s neuroimaging information can be found in Table 2.

**Table 2:** Properties of multimodal images and the reference image for cases included in the collection.

No.	Collected images	Reference image (RI)	Dimension of RI [voxels]	Spacing of RI [mm^3^]
01	T1WI, T1-CE, DTI	T1WI	$224\times 256\times 192$	$0.977\times 0.977\times 1.000$
02	T1WI, T1-CE, T2WI, DTI	T1WI	$192\times 256\times 176$	$0.977\times 0.977\times 1.000$
03	T1WI, T1-CE	T1WI	$192\times 256\times 176$	$0.977\times 0.977\times 1.000$
04	T1WI, T1-CE	T1WI	$192\times 256\times 176$	$0.977\times 0.977\times 1.000$
05	T1WI, T1-CE, DTI	T1WI	$192\times 256\times 176$	$0.977\times 0.977\times 1.000$
06	T1WI, T1-CE, DTI	T1WI	$192\times 256\times 176$	$0.977\times 0.977\times 1.000$
07	T1WI, T1-CE	T1WI	$192\times 256\times 176$	$0.977\times 0.977\times 1.000$
08	T1WI, T1-CE, DTI	T1WI	$192\times 256\times 176$	$0.977\times 0.977\times 1.000$
09	T1WI, T1-CE, DTI	T1WI	$192\times 256\times 176$	$0.977\times 0.977\times 1.000$
10	T1WI, T1-CE, DTI	T1WI	$192\times 256\times 176$	$0.977\times 0.977\times 1.000$
11	T1WI, T1-CE, DTI	T1WI	$192\times 256\times 176$	$0.977\times 0.977\times 1.000$
12	T1WI, T1-CE, DTI	T1WI	$192\times 256\times 176$	$0.977\times 0.977\times 1.000$
13	T1WI, T1-CE, DTI	T1WI	$192\times 256\times 176$	$0.977\times 0.977\times 1.000$
14	T1WI, T1-CE, DTI	T1WI	$192\times 256\times 176$	$0.977\times 0.977\times 1.000$
15	T1WI, T1-CE, DTI	T1WI	$192\times 256\times 176$	$0.977\times 0.977\times 1.000$
16	CT	CT	$512\times 512\times 276$	$0.457\times 0.457\times 0.625$
17	CT	CT	$512\times 512\times 255$	$0.500\times 0.500\times 0.625$
18	CT	CT	$512\times 512\times 310$	$0.621\times 0.621\times 0.625$
19	CT	CT	$512\times 512\times 141$	$0.449\times 0.449\times 1.250$

CT = computed tomography; DTI = Diffusion tensor imaging; T1-CE = T1-weighted contrast-enhanced; T1WI = T1-weighted imaging; T2WI = T2-weighted imaging.

The “General Registration (Elastix)” extension on the 3D Slicer platform facilitated this process^39^. The calculated registration matrix was then saved within the 3D Slicer scene files, enabling the transformation of segmented structures or 3D reconstructed models from multiple modal sequences into the unified coordinate system, thus enhancing the precision and applicability of subsequent analyses and surgical planning.

### Image post-processing

Image post-processing referred to generating model files from volumetric data suitable for 3D printing or holographic visualization. This could be coarsely divided into two main steps: image segmentation and 3D reconstruction (see Fig. 2C and D).

Various segmentations related to the surgical treatment of intracranial lesions were developed, yielding holographic models visualizable through MRN. In neuro-oncological minimally invasive surgical planning, attention was given to the lesion’s location and three-dimensional structure, the segmentation of lesions, adjacent arteries and veins, and functional relevant structures such as major white matter fiber tracts. These structures were deemed significant by surgeons for surgical planning and execution. In the surgical intervention of intracerebral hemorrhage, the segmentation of the hemorrhage’s three-dimensional structure and the models used for surgical guidance (e.g., puncture pathways to the hemorrhage, endoscopic routes, and craniotomy compatible with port surgery) had been delineated. The structures’ segmentation was performed using the “Segment Editor^40^,” “UKF Tractography^41,42^,” “Markups,” and “Curve Maker” extension modules in 3D Slicer software, with the capability for both manual and automatic segmentation. Specifically, structures such as the lesions, the vessels, the hemorrhage, and the ventricles were outlined utilizing automatic segmentation where possible and supplemented by manual adjustments for refinement.

Three-dimensional reconstruction involved layering and aligning segmented two-dimensional images to form a seamless three-dimensional surface, which was essential for holographic visualization or converting the segmented data into a voxel-based format suitable for 3D printing applications (see Fig. 2D). Employing the “Segmentation” and “Model Maker” extensions in the 3D Slicer software, clusters of segmented voxels were converted into detailed 3D models.

### Validation reference objects

**Fig. 3:**
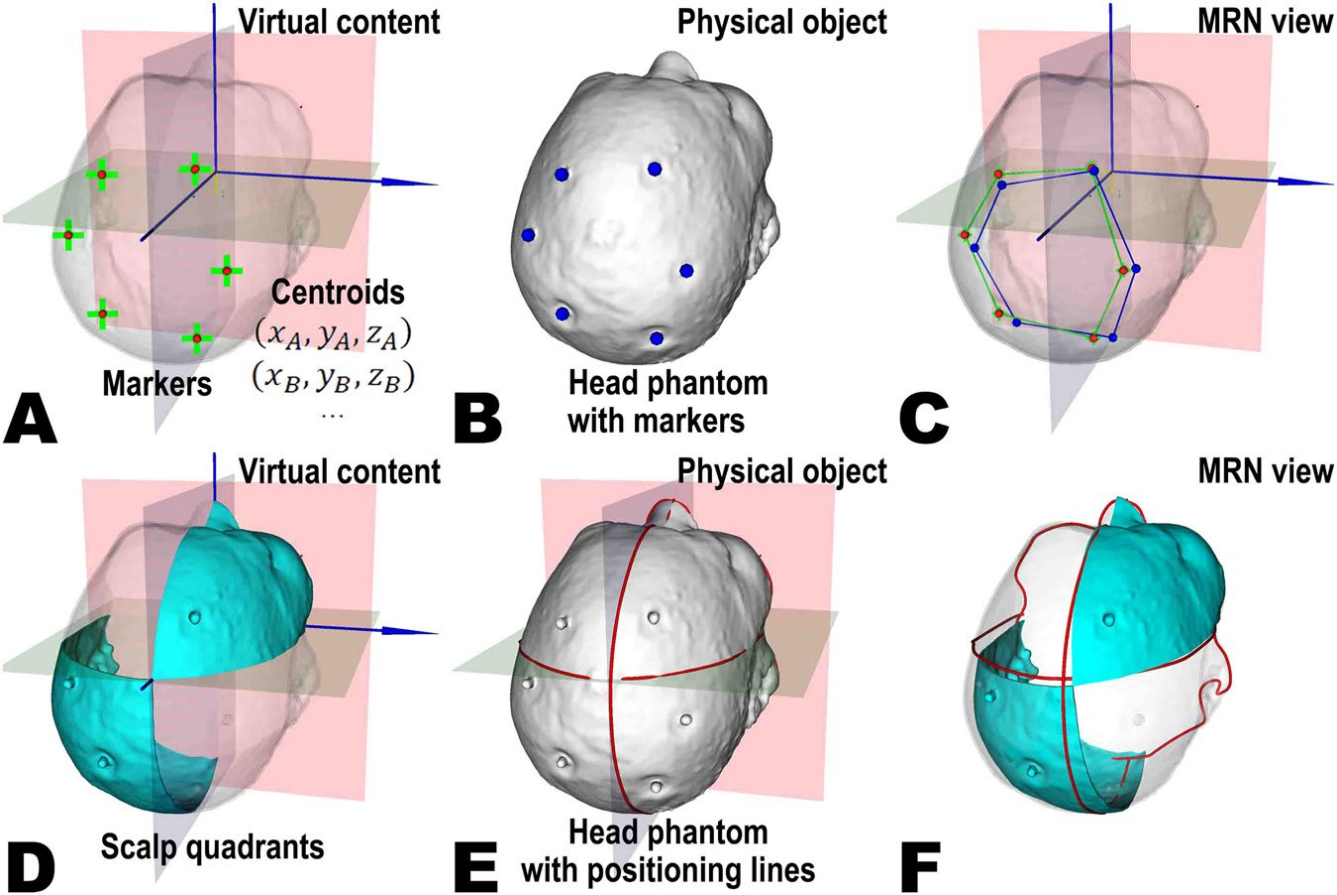
An overview of the validation reference objects principle. Subfigures (**A**–**C**) illustrate the marker-based comparison. In the reference image (RI), centroids (green crosses) of the markers (red spheres) are automatically extracted within the image coordinate system (blue axes) and serve as the ground truth (**A**), while the physical head phantom is designed to incorporate the markers (blue spheres) (**B**). After registering the virtual content to the phantom using the MRN system, the user can capture the coordinates of the perceived physical points (blue spheres) in virtual space, allowing for the measurement of their deviation from the ground truth. Subfigures (**D**–**F**) demonstrate the comparison based on positioning lines. The hologram of scalp quadrants (cyan) is created using orthogonal reference planes and the segmented skin surface from the RI (**D**), while the physical head phantom integrates the laser positioning line models (red lines) (**E**). Once the virtual content is registered to the head phantom with the MRN system, users can observe the mismatch between the scalp quadrant and the physical model of positioning lines, providing an intuitive impression of the registration quality.

Validation reference objects were created to assess the accuracy of MRN systems in aligning virtual images with physical reality. This was achieved by establishing reference objects in virtual and physical space to compare their positional correspondence. Two principal reference relationships were provided in the dataset: (1) Landmark-based comparison, where markers affixed to the patient’s scalp during imaging are identified and segmented, allowing their positions to be visualized in both the MRN system’s virtual images and the physical model (see Fig. 3A–C); (2) laser positioning line comparison, where laser lines projected onto the patient’s skin by the scanner’s frame represented three orthogonal reference planes in the images, corresponding to specific planes in the computer-generated images where the principal axis coordinate value was zero (see Fig. 3D–F). For the implementation, virtual validation objects for import into the MRN system and their corresponding 3D-printed physical models were created. Markers were segmented and modeled, and their centroids were extracted using the “Segment Editor” and “Segment Statistics” extension modules (see Fig. 3A)^40^. Laser positioning lines were modeled using the “Markups” and “Curve Maker” extension modules (see Fig. 3E), with a “Scalp quadrants” virtual model designed via the “Easy Clip” extension module to enhance the visual representation of laser lines in virtual space (see Fig. 3D).

### 3D-printed phantom generation

The STL files used for 3D printing, derived from segmented skin surfaces within reference CT/MRI data, underwent a 3D reconstruction process. This involved a standardized method using the “Segment Editor” extension’s tools (e.g., threshold, paintbrush, scissors, islands, hollowing, and smoothing) to extract a 3D skin surface with a designated thickness of 1 mm^21,35,40^. However, directly using these raw STL files for 3D printing posed several challenges, including surface roughness from noise, discontinuities such as gaps or holes, potentially hazardous sharp spikes or edges from anatomical structures, and an uneven bottom or inclined phantom stance that could complicate the printing process, increase material usage, and extend printing time.

To address these challenges and enhance the continuity of the process from segmentation to printing, the STL file generation was refined for optimal efficiency and quality. The initial step involved applying Gaussian smoothing with a minimal voxel size of approximately 1 × 1 × 1 mm ^3^ during segmentation, significantly reducing surface noise while maintaining anatomical accuracy. Subsequently, a rectangular cropping/filling technique was employed using the “scissor” tool to create a flat bottom surface aligned with the axial standard plane to ensure a stable base for printing. Critical attention was given to smoothing sharp edges to ensure model quality. This comprehensive approach addressed the initial challenges and produced cost-effective, high-quality, and researcher-friendly 3D skin surfaces.

To accommodate various research and testing objectives for MRN systems, two variants of head phantoms were designed by integrating the 3D skin surface with validation reference objects. These variants include one with the 3D skin surface and markers (see Fig. 3B) and another with the 3D skin surface, markers, as well as positioning lines (see Fig. 3E). The integration process was facilitated through the “Merge Models” extension module. Notably, no transformations were applied throughout the generation and optimization of the 3D skin surface, preventing any misalignment with the validation reference objects.

## Data Records

**Fig. 4:**
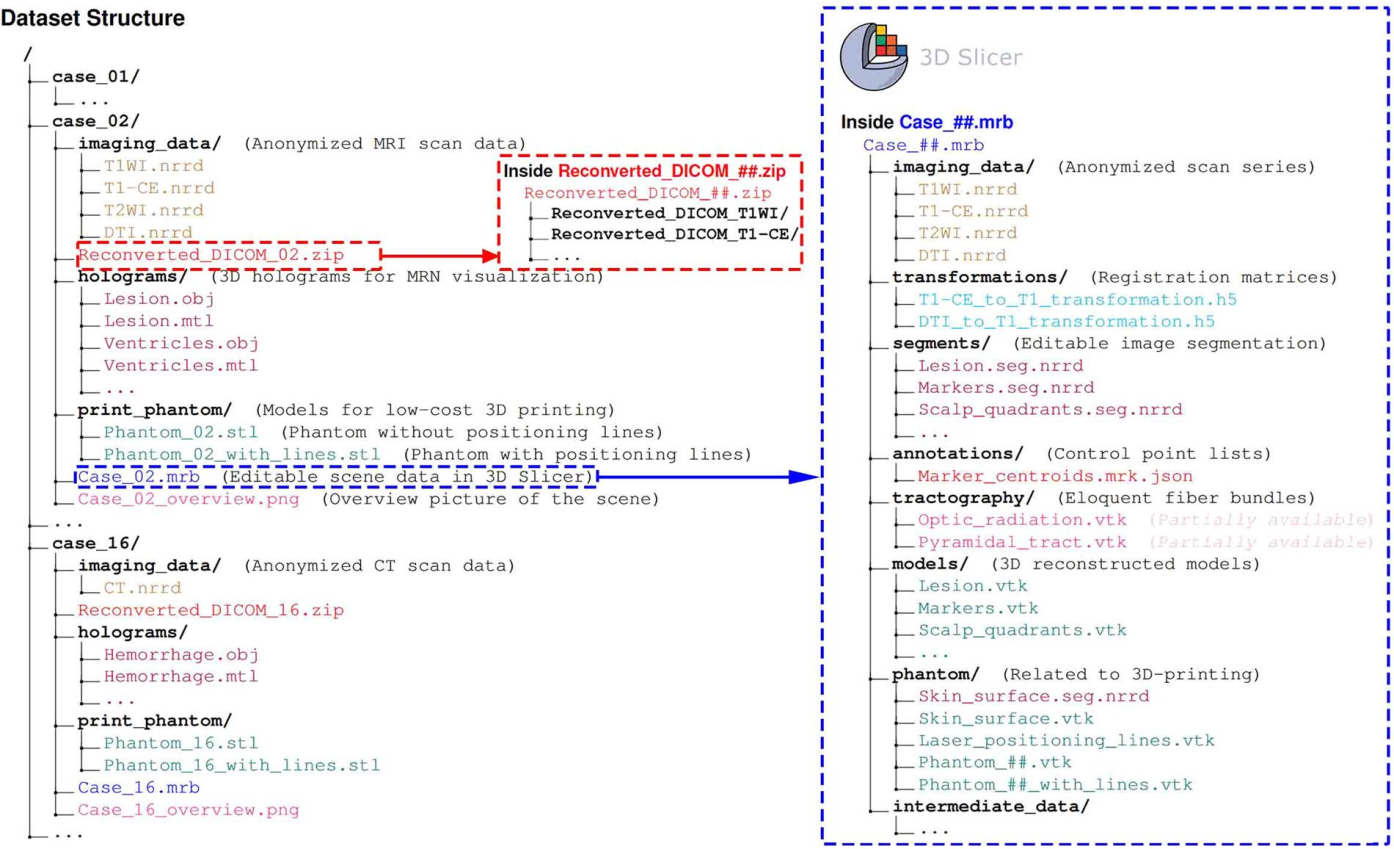
The structure of the dataset (left), an example anonymized DICOM archive file (red box), and an example MRB file (right, blue box). The forward slash “/“ represents a directory.

All imaging data sets and generated meta-data are publicly available at 10.6084/m9.figshare.24550732.v6, stored in FigShare repository^43^. This collection features 47 raw CT/MRI datasets in NRRD and anonymized DICOM archive format from 19 patients, 240 holograms in 133 OBJ files, 19 pairs of STL files (with or without positioning lines) for 3D printing, and 19 scene files in medical reality bundle (MRB) format tailored for processing and generating the aforementioned files within 3D Slicer. Additionally, each case’s marker centroid coordinates are encapsulated within their respective MRB files for precise accuracy assessment and analysis. The data within the dataset is methodically organized into hierarchical directories based on patient ID and file type, exemplified by “case_01” (see Fig. 4). Cross-referencing between the patient IDs in the directory or file names and Tables 1 & 2 in the main manuscript is facilitated. Documented pathological data includes post-operative histopathological results and anatomical location, with lesion volumes automatically calculated via the “Segment statistics” extension and lesion depths determined through the “Model to Model Distance” extension in 3D Slicer (See Table 1). Surgical data encapsulates patient surgical positioning and segmented anatomical structures pertinent to surgical intervention or navigation system co-registration. Voxel and resolution parameters are chronicled in the datasheet for each case’s RI (see Table 2). The 3D printed phantoms’ sizes, material consumption, and anticipated printing durations are reported, enabling researchers to select an appropriate 3D printer and estimate time and financial expenditures (see Table 4).

## Technical Validation

The dataset creation process encompassed four stages: 3D medical imaging, image processing, 3D printing, and the creation of validation objects. Quality control measures were implemented at each stage to ensure rigor and reliability.

### De-identification, anonymization, and integrity of imaging data collection

The CT/MRI scanners used for data collection are certified commercial products routinely employed in clinical settings, operated and maintained by qualified physicians or technicians who also perform regular quality control checks. During the data selection phase, subjects with highly recognizable facial features were excluded, and the non-identifiability of facial characteristics in the retained subjects was confirmed through visual inspection (by Z.Q., X.C., and J.Z.). Subsequently, patient metadata was removed during the data conversion step (from DICOM to NRRD format) to achieve anonymization. Furthermore, each case was visually inspected (by Z.Q.) to ensure that the original imaging data were neither compressed nor corrupted, maintaining the integrity of the dataset.

### Validity and usability of holograms

Image processing was conducted using the open-source platform 3D Slicer to guarantee a replicable model generation process. User-dependent operations, such as segmentation, annotations, and white matter fiber tract reconstruction, were performed by a neurosurgeon (Z.Q., an attending physician with 6 years of experience) with extensive software and neurosurgical expertise. The time required for segmentation operations is detailed in Table 3. Generating data packages for each case of neurological neoplasms took approximately 60 minutes, while for each case of hypertensive cerebral hemorrhage, it took around 40 minutes. The final surgical plans were reached through consensus after discussions within the treatment team, including two independent senior neurosurgeons (X.C. and J.Z., chef physicians with more than 20 years of experience each). In prior MRN studies, the MRN system based on Microsoft HoloLens-2 (Microsoft, Redmond, WA, USA) demonstrated fundamental consistency with co-registered commercial navigation systems, validating the clinical effectiveness of the segmentation process. Specifically, the successful visualization of all 240 holograms substantiates the usability of 133 OBJ files within the MRN system in the previous study^21^.

**Table 3:** Segmentation time for neurological neoplasm and cerebral hemorrhage cases.

Segmentation for neurological neoplasm	Time [minutes]	Segmentation for cerebral hemorrhage	Time [minutes]
Lesion(s)	2	Hemorrhage	2
Ventricles	5	Ventricles	5
Venous sinus	5	Frontal Sinus	5
Arteries	10	Bone flap	5
Pyramidal tract	15	Puncture path	2
Optic radiation	15	Endoscopy path	2
Scalp quadrants	10	Scalp quadrants	10
MRI Markers	2	CT Markers	2
Total time	54*	Total time	33

(*) The whole-brain fiber bundle reconstruction only needs to be performed once (approximately 10 minutes), as it is a shared process for both the pyramidal tract and the optic radiation. Therefore, the total time should be reduced by 10 minutes.

### Validity and usability of 3D-printable head phantoms

To ensure accurate 3D printing of the phantom heads, a commercial 3D printer, A5S (Shenzhen Aurora Technology Co., Ltd, China), was used to create 1:1 scale models for all 19 cases (parameters: nozzle temperature: 210 °C, platform temperature: 50 °C, material: polylactic acid (PLA), resolution: 0.3 mm, fill level: 10%). All 19 models with positioning lines were successfully printed^21^, with an average duration of 22.4 ± 3.1 hours and an average cost significantly lower than commercial head phantoms, demonstrating the process’s efficiency and cost-effectiveness (See Table 4). While models without positioning lines were not individually validated through 3D printing, their simpler design compared to those with positioning lines suggests they could also be successfully printed.

**Table 4:** Properties of 3D-printed phantoms. PLA = polylactic acid; 3D = three-dimensional.

Phantom No.	Size [cm^3^]	Weight [g]	PLA material length [meter]	3D-printing time [hours]	Cost [£]
01	$21.3\times 18.1\times 16.4$	453	151.90	22.6	4.93
02	$18.9\times 22.6\times 15.6$	475	159.32	25.1	5.17
03	$19.5\times 18.7\times 16.9$	417	139.71	21.8	4.54
04	$20.8\times 20.2\times 17.5$	530	177.79	27.8	5.77
05	$18.9\times 21.1\times 15.7$	447	149.98	22.7	4.86
06	$18.0\times 20.2\times 16.7$	406	135.99	21.3	4.42
07	$19.0\times 23.0\times 16.1$	491	164.58	26.3	5.34
08	$16.9\times 19.6\times 13.8$	370	123.96	19.0	4.03
09	$16.9\times 20.1\times 15.7$	352	117.86	19.0	3.83
10	$19.4\times 20.0\times 15.1$	402	134.85	21.2	4.37
11	$17.2\times 19.5\times 15.2$	386	129.57	20.3	4.20
12	$22.4\times 19.7\times 16.0$	509	170.69	25.0	5.54
13	$18.0\times 19.1\times 13.1$	341	114.43	18.7	3.71
14	$18.3\times 21.8\times 14.1$	378	126.87	19.9	4.11
15	$17.1\times 20.0\times 16.6$	375	125.84	20.0	4.10
16	$20.0\times 18.9\times 14.8$	360	120.72	18.6	3.92
17	$18.7\times 22.9\times 16.7$	454	152.22	23.5	4.94
18	$20.2\times 20.7\times 15.5$	534	178.96	27.7	5.81
19	$20.5\times 17.8\times 10.9$	392	131.58	19.1	4.27

### Usability of validation reference objects

Positioning lines and markers were generated using a semi-automated method, whereas the extraction of marker centroids and the calculation of their coordinates were automated, ensuring high reproducibility. In prior research by the study group, positioning lines served as a visual reference for MRN system alignment assessment, and markers were used for quantitative evaluations. Specifically, they acted as known points in space (i.e., the ground truth) to provide references for measured points in experiments, aiding in the calculation of metrics critical for assessing MRN system accuracy, such as fiducial localization error (FLE), fiducial registration error (FRE), and target registration error (TRE). The centroid, virtual point, and physical point coordinates were collected for all markers in the study^21^, accumulating a total of 124 coordinate pairs. Across all measurements, the **FLE** was 1.9 ± 1.0 mm, **TRE** was 3.0 ± 1.1 mm, and **FRE** was 2.1 ± 0.6 mm. Given these outcomes, it’s reasonable to assert that the dataset quantitatively reflects the accuracy of the MRN system. Measurements, albeit user-dependent, are consistently reliable. The geometric congruence between the virtual and physical models is profound, thereby not significantly influencing the accuracy evaluation of the MRN system or analogous systems.

### Dataset scalability

The dataset exhibits commendable scalability. In the context of MRN, scalability pertains to the potential of the dataset to be extrapolated and applied in environments, devices, or algorithms different from the original research scenario, effectively facilitating other researchers in developing and testing their MRN systems. Otherwise, its scalability becomes limited if the dataset solely applies to specific research scenarios. Hence, during dataset creation, this study opted for representative samples and configurations to ensure broad applicability. Firstly, cases encompassed in this dataset span diverse lesion localizations, surgical positions, and neurosurgical intervention plans, ensuring clinical balance and mitigating case selection biases during new system testing. Secondly, this dataset is conducive to validating other MRN or AR systems, e.g., AR systems mounted on smartphones or tablets. As long as researchers integrate quantitative measurement modules (e.g., virtual probes, rulers, or protractors) within their systems, they can conduct quantitative assessments on known marker points based on their requirements. Lastly, the dataset is compatible with various MRN registration methods. For example, known markers on the 3D-printed phantom facilitate research and evaluation of landmark-based registration, while phantoms with and without positioning lines are congruent with LCS registration and surface-based registration. This dataset offers comprehensive generalizability across cases, devices, and algorithms, manifesting technical and economic efficiencies.

## Usage Notes

Any individual or institution may freely download, share, copy, or republish the data in any medium or format for reasonable research purposes. The dataset is licensed under Creative Commons Attribution 4.0 International (CC BY 4.0) (https://creativecommons.org/licenses/by/4.0/). Additionally, our data permits researchers to adapt, adjust, modify, or transform according to their research objectives. We aim to offer minimally user-dependent models in the public dataset, allowing researchers to test and optimize their MRN systems.

### Medical image processing

NRRD is a widely-used file storage format for medical imaging, supported by various free and open-source medical imaging software such as 3D Slicer (https://www.slicer.org/), ITK-SNAP (https://www.itksnap.org/https://www.itksnap.org/), MeVisLab (https://www.mevislab.de/), Studierfenster (www.studierfenster.at), DicomWorks (https://www.dicomworks.com/), etc. It is also supported by programming languages and platforms such as MATLAB (https://www.mathworks.com/), Python (https://www.python.org/), and the VTK (https://www.vtk.org/). Commercial image processing software can further process or analyze the areas or structures of interest.

In this study, the processing of medical images was conducted entirely within the 3D Slicer platform. 3D Slicer is a powerful open-source software platform for medical image processing and computer-assisted surgery. With its robust integrative and modular design^38^, users can select desired extension modules for expansion or integrate renowned external tools and libraries (e.g., VTK, Insight Toolkit (ITK) (http://www.itk.org), Python libraries). Furthermore, 3D Slicer boasts an active developer and user community, providing abundant educational and training resources, significantly enhancing the possibility and flexibility for clinicians and researchers to obtain free support. We encourage clinicians and researchers to customize their medical image processing methodologies, data, and models using the 3D Slicer platform as per their requirements. To facilitate this, well-organized MRB files are provided for each case in the dataset. MRB, a binary format, encapsulates all data within a 3D Slicer scene and is directly supported by the 3D Slicer software. Moreover, it can be transformed into a .zip file by simply changing the extension, allowing users direct access to the internal data.

### Holographic visualization & 3D printing

OBJ and STL are widely accepted standard file formats in the 3D graphics industry, gaining popularity among many 3D modeling and computer graphics communities due to their simplicity, flexibility, and extensive support. In the dataset, it’s noteworthy that each OBJ file is accompanied by a corresponding material library (MTL) file within the same folder. The MTL is a ubiquitous file format that applies color and material information to the OBJ files, allowing researchers to open and use the OBJ files more quickly and conveniently. There are numerous platforms and libraries supporting OBJ and STL, including but not limited to, open-source platforms such as 3D Slicer, CloudCompare (https://www.cloudcompare.org/main.html), Blender (https://www.blender.org/), Three.js (https://threejs.org/), commercial platforms such as AutoCAD (https://www.autodesk.com/), Maya (https://www.autodesk.com/products/maya/overview), 3ds Max (https://www.autodesk.com/products/3ds-max/overview), Cinema 4D (https://www.maxon.net/en/cinema-4d), and those in between such as Unity (https://www.unity.comhttps://unity.com), SketchUp (https://www.sketchup.com), and Unreal Engine (https://www.unrealengine.com/). Users can choose their desired platform for further editing or rendering based on their needs. In the context of MRN system development and testing, OBJ and STL are natively supported files by mainstream MR HMDs, allowing direct importation and visualization without further operations. Additionally, most commercial 3D printer software platforms support the STL format, making the provided STL in this dataset directly usable for printing.

## Data Availability

The creation of the dataset was entirely based on the open-source software platform, 3D Slicer, without the use of custom code.
